# Second row of eyelashes with lower extremity edema

**DOI:** 10.1016/j.jdcr.2023.06.023

**Published:** 2023-06-26

**Authors:** Lauryn Orsillo, Justice Brown, Sara Yumeen, Oliver Wisco, Matthew Clark

**Affiliations:** a Western University of Health Sciences, College of Osteopathic Medicine of the Pacific - Northwest, Lebanon, Oregon; b Department of Dermatology, Warren Alpert Medical School of Brown University, Providence, Rhode Island; c Dermatology Health Specialists, Bend, Oregon

**Keywords:** distichiasis, distichiasis syndrome, lymphedema

## History

**Fig 1:**
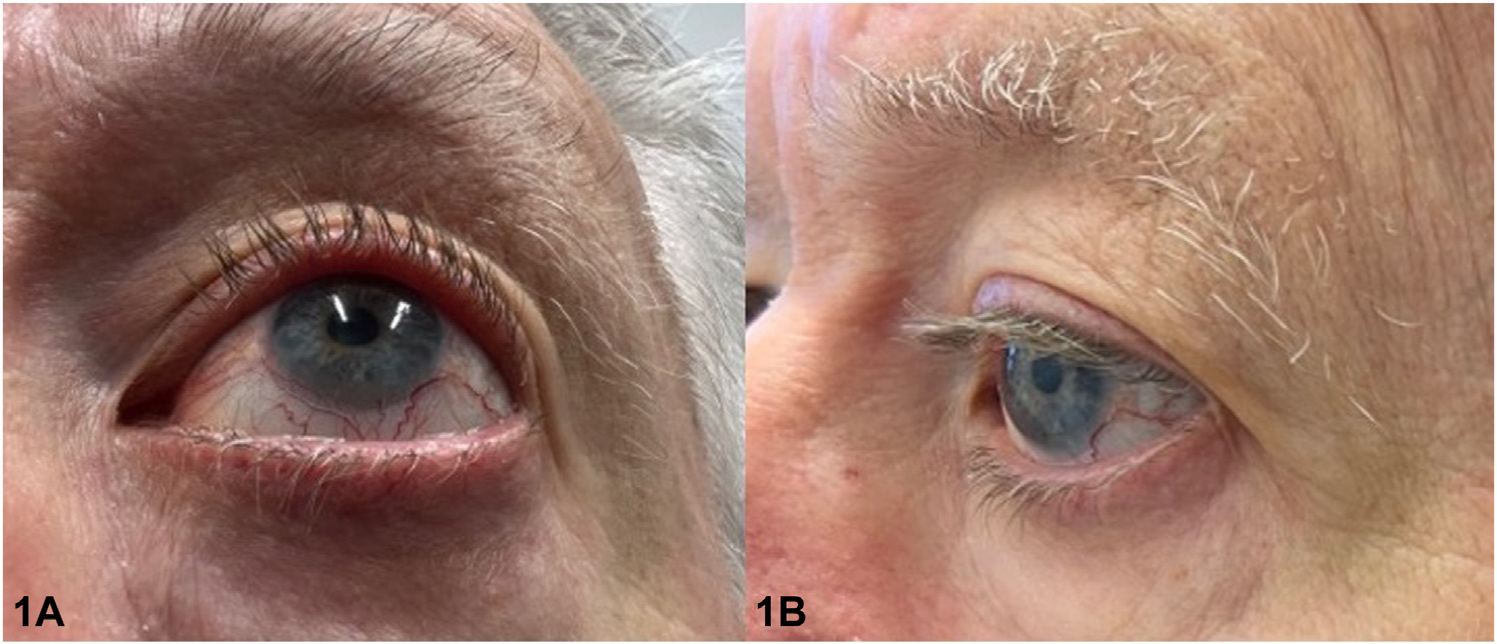

**Fig 2:**
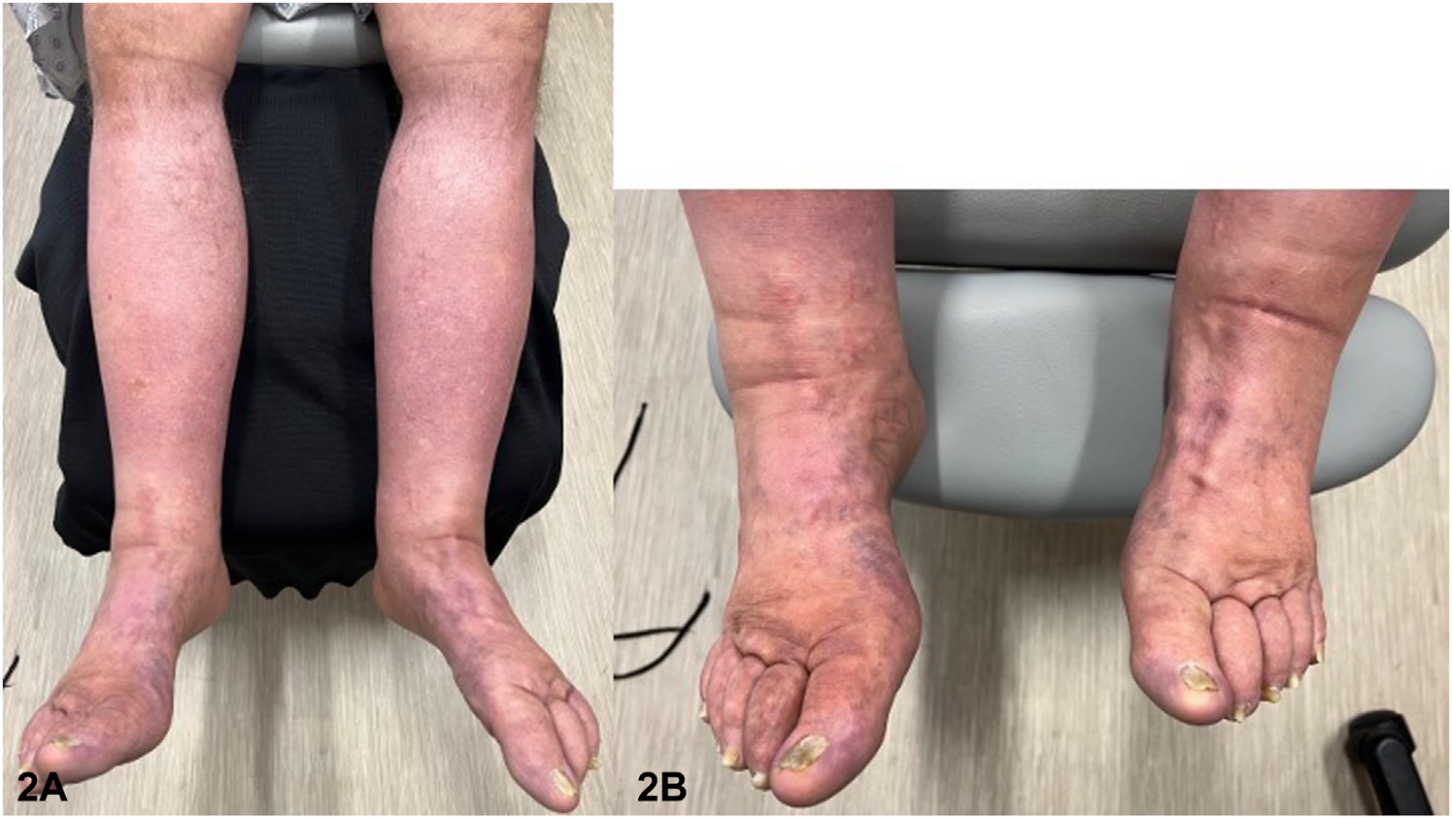

A 71-year-old male presented for a full-body skin examination due to a history of basal cell and squamous cell carcinoma. Physical examination revealed a second row of eyelashes emerging from the meibomian gland on bilateral upper and lower eyelids, which the patient stated had been present his entire life (Fig 1). No trichiasis was noted. Further inspection showed pitting edema with mild fibrosis in the bilateral lower extremities (Fig 2). The patient stated that his daughter had lower extremity lymphedema and distichiasis as well.

**Question 1: What is the most likely diagnosis?**

A. Ocular Cicatricial Pemphigoid
B. Lymphedema Distichiasis syndrome (LDS)
C. Pierre Robin syndrome (PRS)
D. Milroy disease
E. Setleis syndrome

**Answers:**

A. Ocular Cicatricial Pemphigoid – Incorrect. This is a mucous membrane pemphigoid characterized by bilateral conjunctivitis. Distichiasis in this condition is acquired and is not associated with lymphedema.
B. LDS – Correct. The patient's presentation is consistent with LDS. LDS is a rare condition in which patients develop lower extremity lymphedema and a second row of eyelashes emerging from what would have been the meibomian glands (distichiasis).^1^ Distichiasis affects 94% to 100% of patients and is often present at birth.^2^^,^^3^ While it can be asymptomatic, in 75% of cases it causes chronic keratitis, conjunctivitis, and photophobia.^2^ Lymphedema involving the lower extremities and external genitals can affect up to 80% of patients and typically develops during late childhood or puberty.^1^ Diagnosis can be made clinically or through genetic testing for a *FOXC2* mutation.
C. PRS – Incorrect. While this can be rarely associated with LDS (approximately 0.5% of PRS cases), both conditions more often present separately. Classic findings, including micrognathia, cleft palate, and tongue displacement, were not present.
D. Milroy disease – Incorrect. It can also present with lower extremity lymphedema and atypical eyelashes, although lymphedema is usually present at or near birth.
E. Setleis syndrome – Incorrect. Distichiasis can be a manifestation of Setleis syndrome. However, this patient did not present with the characteristic bitemporal scar-like lesions. Infants may be missing eyelashes on both upper and lower lids or have multiple rows of lashes on the upper lids and none on the lower.

**Question 2: Mutation in which gene causes this condition?**

A. DQB1∗0301
B. TWIST2
C. FLT4
D. FOXC2
E. SOX18

**Answers:**

A. DQB1∗0301 – Incorrect. DQB1∗0301 is associated with Ocular Cicatricial Pemphigoid, characterized by bilateral conjunctivitis, and can present with acquired distichiasis.
B. TWIST2 – Incorrect. Mutations in TWIST2 cause Setleis syndrome. It inhibits the expression of target genes involved in dermal and bone development.
C. FLT4 – Incorrect. FLT4 mutations are associated with Milroy disease. This gene is a transmembrane receptor for vascular endothelial growth factor C and vascular endothelial growth factor D.
D. FOXC2 – Correct. Loss-of-function mutations in the *FOXC2* gene cause LDS. It is inherited in an autosomal dominant manner and 75% of those with LDS have an affected parent.^3^ This gene plays a role in embryogenesis and the development of lymphatic vessels, veins, lungs, cardiovascular system, and kidneys.^2^ When mutated, it prevents the development of lymphatic valves and increases recruitment of mural cells to lymphatic capillaries, resulting in insufficient movement of lymphatic fluid and subsequent lymphedema. Additionally, it is highly expressed in venous valves leading to venous insufficiency and varicose veins when mutated.^4^ While the pathogenesis of distichiasis remains unclear, mutations have been shown to interfere with the interaction of the FOXC2 protein with the *Wnt4* promoter, which has been hypothesized to result in abnormal signaling from the Wnt4-Frizzled-RYK signaling pathway. This may cause the abnormal differentiation of the meibomian gland into hair follicles, leading to distichiasis.^5^
E. SOX18 – Incorrect. Variants in the SOX18 gene cause hypotrichosis-lymphedema-telangiectasia. This gene plays a role in the development of the lymphatic system.

**Question 3: How do you treat congenital distichiasis?**

A. Intense Pulsed Light (IPL)
B. Warm Compress
C. Electrolysis
D. Topical Cyclosporine
E. Monitor

**Answers:**

A. IPL – Incorrect. IPL is used to treat Meibomian gland dysfunction which can lead to acquired distichiasis. IPL is thought to increase the skin temperature of the eyelid making the meibum less viscous, unclogging the gland. It also helps to reduce inflammation and reduce risk of infection.
B. Warm Compress – Incorrect. A warm compress can be used to treat blepharitis which can lead to acquired distichiasis. It increases circulation and helps to increase secretion production from the meibomian glands, however it would not treat the distichiasis itself.
C. Electrolysis – Correct. While distichiasis is congenital in LDS, several other variants of distichiasis can be acquired. Distichiasis in both congenital and acquired forms are treated in similar fashions, with both surgical and nonsurgical approaches to treatment, which is often aimed at removal of the second row of eyelashes, as they can cause chronic trauma and inflammation to the conjunctiva. Surgical options include partial tarsal plate excision, wedge resection, and palpebral conjunctival resection.^3^ Nonsurgical options include electrolysis, epilation, and cryotherapy.
D. Topical Cyclosporine – Incorrect. Topical cyclosporine is used in individuals with blepharitis who have not responded to standard treatments. It increases meibomian gland expressibility and tear break up time.
E. Monitor – Incorrect. While not a definitive treatment for congenital distichiasis, monitoring an asymptomatic patient for eye irritation is acceptable management.

## Conflicts of interest

None disclosed.
